# Nonlinear dynamics of postural control system under visual-vestibular habituation balance practice: evidence from EEG, EMG and center of pressure signals

**DOI:** 10.3389/fnhum.2024.1371648

**Published:** 2024-04-26

**Authors:** Anke Hua, Guozheng Wang, Jingyuan Bai, Zengming Hao, Jun Liu, Jun Meng, Jian Wang

**Affiliations:** ^1^Department of Sports Science, Zhejiang University, Hangzhou, China; ^2^Sciences Cognitives et Sciences Affectives, University of Lille, Lille, France; ^3^College of Biomedical Engineering and Instrument Science, Zhejiang University, Hangzhou, China; ^4^Taizhou Key Laboratory of Medical Devices and Advanced Materials, Research Institute of Zhejiang University, Taizhou, China; ^5^Department of Rehabilitation Medicine, First Affiliated Hospital, Sun Yat-sen University, Guangzhou, China; ^6^College of Control Science and Engineering, Zhejiang University, Hangzhou, China; ^7^Center for Psychological Science, Zhejiang University, Hangzhou, China

**Keywords:** postural control, visual-vestibular conflict, fractal dimension, complexity, source localization

## Abstract

Human postural control system is inherently complex with nonlinear interaction among multiple subsystems. Accordingly, such postural control system has the flexibility in adaptation to complex environments. Previous studies applied complexity-based methods to analyze center of pressure (COP) to explore nonlinear dynamics of postural sway under changing environments, but direct evidence from central nervous system or muscular system is limited in the existing literature. Therefore, we assessed the fractal dimension of COP, surface electromyographic (sEMG) and electroencephalogram (EEG) signals under visual-vestibular habituation balance practice. We combined a rotating platform and a virtual reality headset to present visual-vestibular congruent or incongruent conditions. We asked participants to undergo repeated exposure to either congruent (*n* = 14) or incongruent condition (*n* = 13) five times while maintaining balance. We found repeated practice under both congruent and incongruent conditions increased the complexity of high-frequency (0.5–20 Hz) component of COP data and the complexity of sEMG data from tibialis anterior muscle. In contrast, repeated practice under conflicts decreased the complexity of low-frequency (<0.5 Hz) component of COP data and the complexity of EEG data of parietal and occipital lobes, while repeated practice under congruent environment decreased the complexity of EEG data of parietal and temporal lobes. These results suggested nonlinear dynamics of cortical activity differed after balance practice under congruent and incongruent environments. Also, we found a positive correlation (1) between the complexity of high-frequency component of COP and the complexity of sEMG signals from calf muscles, and (2) between the complexity of low-frequency component of COP and the complexity of EEG signals. These results suggested the low- or high-component of COP might be related to central or muscular adjustment of postural control, respectively.

## 1 Introduction

Individuals need to constantly maintain upright standing balance, responding to complex and dynamically changing environments. This intricate process is known as postural control. The dynamics of human postural control are inherently complex (Ting et al., 2009; Ivanenko and Gurfinkel, 2018), and such complexity of the postural control system arises from the nonlinear interaction among multiple subsystems over multiple time scales, including musculoskeletal, sensory and neural systems (Shumway-Cook and Woollacott, 2014).

Thus, recent studies applied nonlinear methods [such as entropy, fractal dimension (FD) and recurrence quantification analysis] to explore the dynamic characteristics of center of pressure (COP) oscillations, as an important measure of postural sway, when facing different perturbations [see the review (Kędziorek and Blażkiewicz, 2020)]. Physically speaking, higher value of entropy or FD reflects increased complexity of time series. In context of postural control, when facing perturbations such as vibrations on the calf, the complexity of COP oscillations in both young and older participants decreased initially before gradually increasing with such sustained vibrations (van den Hoorn et al., 2018). Also, absence of visual information in young and older participants decreased the complexity of COP data (Ramdani et al., 2011). The results suggested that the lower complexity of sway translates into lower flexibility of postural control, and the increased complexity interpreted as improved self-organization and effective strategies in postural control (Kędziorek and Blażkiewicz, 2020). Furthermore, numbers of studies used these complexity-based nonlinear methods to study the decrease in postural stability caused by aging, and suggested that, compared to young adults, the decreased postural sway complexity among elderly people reflected lower flexibility and adaptive capacity of postural control system (Manor et al., 2010; Zhou et al., 2017; van den Hoorn et al., 2018). These results are consistent with the theory of complexity loss in aging, suggesting that the adaptive abilities reduce with aging (Goldberger et al., 2002).

**Figure 1:**
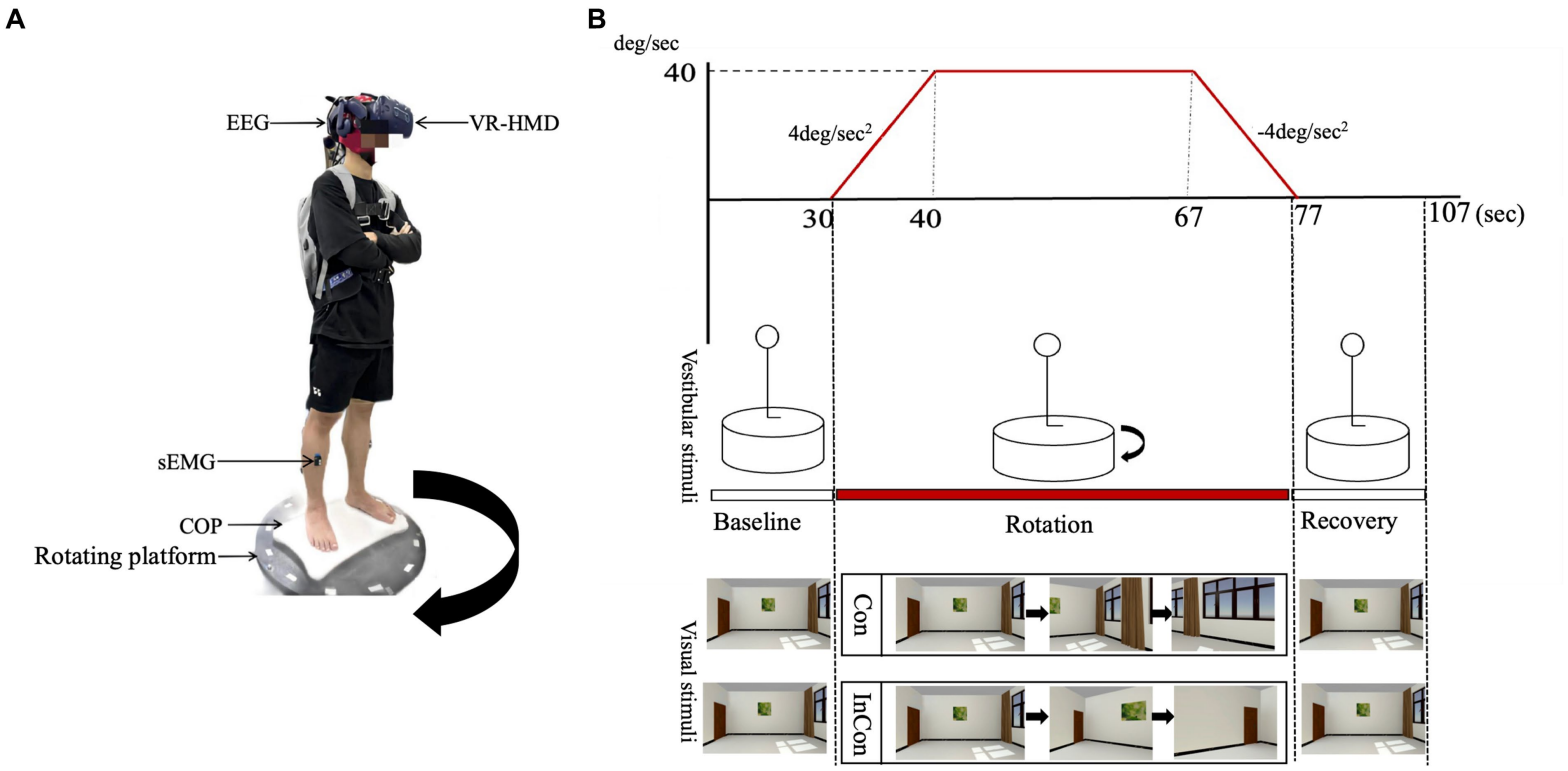
**(A)** An illustration of the laboratory instruments used in the present experiment. The direction of the rotating platform was clockwise. **(B)** An illustration of the visual scene and the rotating platform. The visual scene and the rotating platform was synchronized through the Unity3D program in the present study. The visual scene in the VR headset was rotated counterclockwise in the congruent condition (Con) (i.e., natural visual environment) and clockwise in the incongruent condition (InCon) (i.e., conflicting visual environment).

Previous studies typically perturbed single sensory inputs to examine their effects on multisensory inputs for postural sway. For example, recent studies used virtual reality (VR) technology to create different visual flow patterns and found that the visual flow in VR induced postural instability and activated neuromuscular regulation in postural control (Kabbaligere et al., 2017; Luo et al., 2018). Besides, VR has also been used to understand how the brain select and organize multiple sensory information (Wright et al., 2014), since VR allows us to manipulate two sensory inputs synchronously. For example Nguyen et al. (2020) used a rotary chair for vestibular manipulation and used a visual moving scene for visual manipulation to present the visual-vestibular conflict when sitting. However, the effect of such visual-vestibular conflict on standing postural control still remains unclear. Thus, our present study combined a rotary platform and a visual rotary scene in the VR headset to present visual-vestibular conflicts (Figure 1).

Although postural control system consists of multiple subsystems, previous studies did not explore how the dynamics of neural or muscular system adapt to complex environments such as the sensory conflicting environment to maintain balance. For example, the central nervous system and the muscular system are both fundamental for postural control; thus, we can use electroencephalography (EEG) and electromyography (EMG) to underline the different cortical and muscular activities under complex environments (Merletti et al., 2010; Edmunds et al., 2019; Barollo et al., 2022; Stehle et al., 2022).

Therefore, our first aim was to investigate the dynamic characteristics of subsystems of postural control system during repeated balance practice under visual-vestibular congruent or incongruent environments in conjunction with EEG, surface EMG (sEMG) and COP signals. Previous studies found that the complexity of EEG data was lower in standing than walking in response to a perturbation (Pakniyat and Namazi, 2021), which suggested that the lower EEG complexity in the frontal lobe may indicate a lower cognitive load or a lower attention level (Ke et al., 2014; Gupta et al., 2021). Accordingly, we hypothesized that the complexity of EEG data would decrease after repeated balance practice. Previous studies found that when the difficulty in balance tasks increased, the lower limb muscle activity increased (Gebel et al., 2019) and the complexity of sEMG data from calf muscles decreased (Murillo et al., 2012; Pakniyat and Namazi, 2021). Since these results suggested that higher sEMG complexity probably reflect an effective strategy in postural muscles when facing perturbations, we hypothesized that the complexity of sEMG data of calf muscles would increase after repeated balance practice.

On the other hand, the dynamics of COP oscillations reflected the activity of different neuromuscular components during postural control in different time scales (Manor et al., 2010; Federolf et al., 2015), as suggested in previous studies where low- and high-frequency variations of COP might be related to central adjustments and peripheral adjustments, respectively (Zatsiorsky and Duarte, 1999; Tahayori et al., 2012). Furthermore, recent studies analyzed the nonlinear dynamics of low- and high-frequency components of COP oscillations when performing different levels of task difficulty and found that the complexity of low-frequency component of COP was related to a better task performance, rather than high-frequency component of COP (Caballero Sánchez et al., 2016; Moreno et al., 2022). These results further explore the potential relationship between complexity of COP and neuromuscular adjustments. However, these studies did not explore the potential relationship between low- or high-components of COP and neuromuscular adjustments based on the sEMG or EEG data.

Thus, our second aim was to investigate whether the dynamics of low- and high-frequency COP oscillations differ since previous studies suggested that low- and high-frequency components of COP might be related to peripheral adjustments (i.e., evidence from EMG signals) or central adjustments (i.e., evidence from EEG signals) (Zatsiorsky and Duarte, 1999; Moreno et al., 2022). We hypothesized that the complexity of low-frequency COP data would decrease while the complexity of high-frequency components of COP data would increase after repeated balance practice. We also hypothesized that the complexity of low- or high-frequency components of COP data would be positively related with the complexity of EEG or sEMG data, respectively.

## 2 Materials and methods

### 2.1 Participants

We used F-test model with ANOVA in G*Power software (Version 3.1 for Mac) to calculate the sample size with a significant level of 0.05, an effect size of 0.25 and a statistical power of 0.8. The total required sample size was 24. Thirty-one university students participated in this study, and were randomly divided into two groups (i.e., congruent or incongruent). All participants had no neurological, skeletal, or muscular problems with normal or corrected vision. They signed a written informed consent form before the experiment. The Ethics Committee of Zhejiang University Psychological Science Research Center permitted our experiment.

The data from one participant in the incongruent group was excluded because of stepping during the experiment. Also, the data from three participants was excluded due to the severe body swaying (i.e., spread their arms or use the hip strategy) during the experiment (one in incongruent group, two in congruent group). Therefore, 14 participants (5 females) were included in the congruent group (mean age 22.93 ± 2.28 years, height 172.21 ± 8.80 cm, body mass 64.78 ± 11.24 kg) and 13 participants (6 females) were included in the incongruent group (mean age 24.85 ± 2.57 years, height 169.35 ± 7.05 cm, body mass 63.38 ± 12.45 kg) for the present study.

### 2.2 Procedure

We combined a rotating platform and a VR headset with the visual scene to manipulate vestibular and visual inputs, respectively. Also, we used the Unity3D program to synchronize the control of the rotating platform and the visual scene to set “congruent” (i.e., natural visual stimulation) and “incongruent” (i.e., conflicted visual stimulation) experimental conditions (Figure 1).

In the congruent condition, the rotating platform was accelerated at 4°/s^2^ for 10s to the right (clockwise) and kept rotating at 40°/s for 27 s, and then decelerated at 4°/s^2^ for 10s, with no additional control in the visual scene. Thus, the visual scene moved counterclockwise relative to the participant. In the incongruent condition, the rotating platform was set to the same parameters as in the congruent condition. In this case, the visual scene moved clockwise relative to the participant, providing visual information that was opposite to the actual motion (Figure 1).

Before the experiment, demographic data (e.g., age, height, body mass, dominant side) was recorded for each participant. Participants were asked to stand on the stable platform and to cross their arms on the chest for 30 s (baseline) followed by 47 s of “congruent” or “incongruent” condition (rotation) and finally again on the stable platform for 30 s (recovery). Each participant was asked to complete five repeated tasks, either congruent or incongruent, with 5-min rest between each standing task. During the standing task, each participant was asked to cross their arms on the chest.

### 2.3 Data collection

We recorded COP data, surface EMG (sEMG) data and EEG data from participants during the experimental tasks. A Wii balance board was placed in the center of the rotating platform and the BrainBlox program software was used to collect COP data with a 100 Hz sample frequency (Cooper et al., 2014). A 16-channel sEMG system (Trigno Wireless System, Delsys, United States) was used to collect the sEMG data of the left and right Tibialis anterior (TA) and Medial gastrocnemius (MG) with a 2,000 Hz sample frequency. The skin was shaven, abraded, and cleaned with alcohol prior to placing the electrodes. A double-sided tape was used to fix the electrodes. Also, an ANTNeuro EEG device containing 32 channels in the 10–20 standard regime was used to collect EEG data. The impedance of all electrodes remained below 5 k ohms throughout the experiment with a 1,000 Hz sampling frequency.

### 2.4 Data analysis

In the present study, we used the rotating platform to manipulate the vestibular information since the semicircular canals within the vestibular system are responsible for detecting the angular acceleration information. Thus, we divided raw data of COP, sEMG and EEG into two phases for further analysis: acceleration (0–10 s after platform start) and deceleration (37–47 s after platform start).

Since the postural control system is inherently dynamically nonlinear (Ivanenko and Gurfinkel, 2018) and previous studies showed that COP, sEMG, and EEG signals all have the positive Lyapunov exponent (Sbriccoli et al., 2001; Kannathal et al., 2005; Liu et al., 2015), complexity-based nonlinear methods can be used to identify mechanisms underlying variability in postural control (Ting et al., 2009; Kędziorek and Blażkiewicz, 2020). In the present study, fractal dimension (FD) analysis with the Higuchi’s algorithm was used for providing an indication of the complexity of a signal and quantifying the self-similarity of a pattern over multiple time-scale (Higuchi, 1988). Higher FD value suggest higher complexity of time series. Higuchi’s algorithm for FD calculation was shown below (Cui et al., 2017): first, construct *k* new signals from a given COP signal to a newly constructed signal $S_{m}^{k}$ ; then, calculate the length $L_{m}\left(k\right)$ of $S_{m}^{k}$ and compute the mean of $L_{m}\left(k\right)$ over *m* called *L(k)*; finally, plot *L(k)* against *k* (ranging from 1 to $k_{\max}$ ) on a double logarithmic scale and calculate the slope of this line as the FD index. To choose an appropriate $k_{\max}$ , each FD index was plotted against the $k_{\max}$ . The point at which the plateaus is considered a saturation point, and the $k_{\max}$ should be chosen (Doyle et al., 2004).

To better understand the linear and nonlinear interactions of postural control system, we also calculated the traditional linear measures of COP, sEMG and EEG signals as complementary analysis.

#### 2.4.1 COP data

Recorded COP data were processed offline using a custom script in MATLAB (R2021a, MathWorks, United States). A filtering procedure was used as a method of decomposing the COP data (1,000 points of acceleration or deceleration) into two different components to reveal the underlying mechanisms of postural control. A 0.5 Hz low-pass Butterworth filter (4th-order, zero-phase lag) was used to obtain the low-frequency component of the COP data (Zatsiorsky and Duarte, 1999; Moreno et al., 2022). A 0.5–20 Hz band-pass Butterworth filter (4th-order, zero-phase lag) was used to obtain the high-frequency component of the COP data (Zhou et al., 2017). We also filtered COP data through a 20 Hz low-pass Butterworth filter (4th-order, zero-phase lag) and calculated the standard deviation (SD). The outcome measures for COP data used in this study were the FD value of low-frequency and high-frequency components of COP time series and the SD value in the AP and ML directions.

Previous studies applied nonlinear methods to explore the dynamic characteristics of COP signals in response to various perturbations or across different groups, such as young people, elderly people with or without fall history, and they generally low-pass filtered the raw COP data with a cutoff frequency at 20 Hz (Luo et al., 2018; van den Hoorn et al., 2018; Hao et al., 2021). It has been found that perturbations decreased the complexity of COP data (i.e., lower FD value and lower entropy value), with a more significant effect observed in elderly individuals (Manor et al., 2010; Zhou et al., 2017; Kędziorek and Blażkiewicz, 2020). Lipsitz (2002) proposed a “loss of complexity” hypothesis to study the decrease in physiology function caused by aging. Accordingly, higher complexity of COP signal might suggest higher flexibility of postural control system and an effective postural strategy used in postural control.

#### 2.4.2 sEMG data

Recorded sEMG data were processed offline using a custom script in MATLAB (R2021a, MathWorks, United States). We used a 20–500 Hz band-pass, 4th-order, zero-lag Butterworth filter. Then, the 50 Hz line noise was removed. We used the filtered sEMG data (20,000 points of acceleration or deceleration) to calculated the integrated EMG (iEMG), and normalized iEMG value to the baseline activity of 5th trial (5 s–15 s after experiment start) among muscles and across participants as normalized muscle activity. The outcome measures for sEMG data used in this study were the FD value of the time series and the normalized muscle activity of left and right TA and MG muscles.

The majority of previous studies using nonlinear methods on sEMG signals are related with muscle fatigue. For example, it has been reported that muscle fatigue decreased the complexity of muscle sEMG data (i.e., lower FD value and lower entropy value) (Rampichini et al., 2020). Besides, Murillo et al. (2012) found that the complexity of calf muscle sEMG decreased (i.e., lower entropy value) when standing on a more unstable surface. Accordingly, higher complexity of sEMG signal might suggest that muscles are more likely to respond flexibly to a perturbation.

#### 2.4.3 EEG data

Recorded EEG data were processed offline using a custom script in MATLAB (R2021a, MathWorks, USA), and were processed based on a custom script in the EEGLAB toolbox (Delorme and Makeig, 2004).

First, the raw EEG data were filtered with a 1–48 Hz band-pass FIR filter and the 50 Hz line noise was removed using the EEGLAB Cleanline plug-in. The average of all electrodes was chosen as the reference. Further, to remove artifacts from body motion during rotation, the EEG data segments contaminated with large artifacts were removed using Artifact Subspace Reconstruction (ASR) (Mullen et al., 2015), where the threshold was set to 20 standard deviations (Chang et al., 2019) and ensured that at least 80 percent (Every 10s data is guaranteed to leave 8 s) of the data were retained. Finally, the EEG signal was decomposed using independent component analysis (ICA) with the aid of the ICLabel plug-in to remove interfering signals such as blinks, muscle artifacts, electrocardiogram, and linear noise that are not homologous to the EEG (Pion-Tonachini et al., 2019). No baseline removal procedures were performed on the EEG data in the present study.

Adapting from our previously published work (Wang et al., 2022), a standardized low- resolution EEG tomography software package (sLORETA) for source localization (Pascual-Marqui, 2002). Specifically, we selected the following seven cortical regions as the regions of interest (ROIs) defined by the Brodmann atlas: dorsolateral prefrontal cortex (DL-PFC; BA10, 46, 47), frontal eye field cortex (FEF; BA8, 9), motor cortex (MC; BA4, 6), primary somatosensory (S1; BA1, 2, 3), posterior parietal cortex (PPC; BA5, 7), temporal–parietal junction (TPJ; BA22, 40), visual cortex (VC; BA17, 18, 19).

We used the fast Fourier transformation (FFT) analysis with a 10% Hanning window to calculate the spectral power values at alpha band (8–12 Hz) for seven ROIs. The alpha-band power values were normalized to the spectral power values at 4–45 Hz band among ROIs and across participants. The outcome measures for EEG data used in this study were the FD value of the time series and normalized alpha-band power values of seven cortical ROIs.

Previous studies suggested that the complexity of EEG increased (i.e., higher entropy value) when the attention level increased (Ming et al., 2009; Ke et al., 2014). Furthermore, Gupta et al. (2021) found that the meditation reduced the complexity of EEG (i.e., lower FD value), suggesting the brain improved the ability for effectively handling cognitive load. When there was a pull perturbation, the complexity of EEG signals was higher (i.e., higher FD value and higher entropy value) in walking than standing (Pakniyat and Namazi, 2021). Accordingly, lower complexity of EEG signal might suggest that the brain is more likely to effectively handle cognitive load or to deal with complex environments.

### 2.5 Statistical analysis

Statistical analysis was performed using SPSS software (Version 24.0 for Mac). We combined the dependent variables from acceleration and deceleration phases for the further statistical analysis. Differences in measures were assessed through a mixed two-way ANOVA to examine the effect of practice (1st and 5th trial) and the group (Congruent and Incongruent). A series of *post-hoc* multiple comparisons with Bonferroni correction was used. To investigate the potential relationships between low- or high-frequency components of COP data and central adjustments (based on EEG data) or peripheral adjustments (based on sEMG data) respectively, we used two-tailed Pearson correlations between the complexity of low-frequency components of COP data and the complexity of EEG data, and between the complexity of high-frequency components of COP data and the complexity of sEMG data. Statistical significance was set at *p* < 0.05.

## 3 Results

### 3.1 Measures of COP

**Figure 2:**
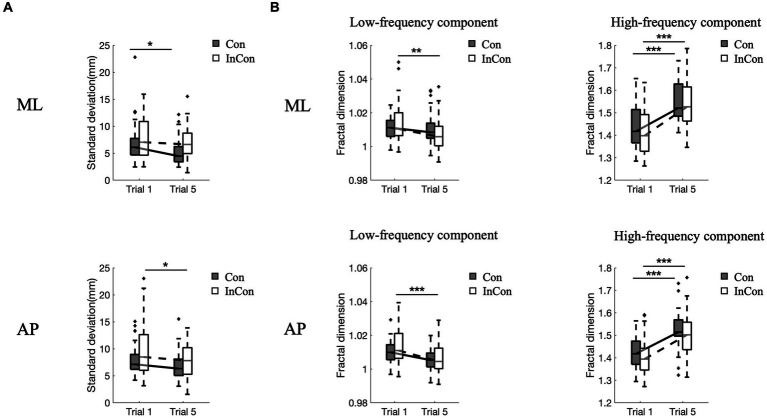
Changes of standard deviation **(A)** and fractal dimension values of low- and high-frequency components of COP data **(B)** in the ML direction and AP direction. In each box plot, the box represents 1st and 3rd quartiles with the median value. The asterisk (*) indicates a significant difference (*<0.05, **<0.01, ***<0.001) between trials. Con and lnC0n represent congruent and incongruent conditions, respectively. AP, anterior–posterior; ML, medial-lateral.

A two-way mixed ANOVA revealed a significant main effect of practice on the FD values of low- and high-frequency components of COP and on the SD values of COP in the AP and ML directions (Supplementary Table S1). However, no significant main effect of group or interaction effect of practice × group was observed. *Post-hoc* tests revealed that practice under the incongruent condition decreased FD_low_ values of COP in the AP and ML directions (*p* < 0.01) (Figure 2B), and decreased SD values of COP in the AP direction (*p* < 0.05) (Figure 2A). Practice under the congruent condition decreased SD values of COP in the ML direction (*p* < 0.05) (Figure 2A). Practice under congruent and incongruent conditions both increased FD_high_ values of COP in the AP and ML directions (*p* < 0.001) (Figure 2B).

### 3.2 Measures of sEMG

**Figure 3:**
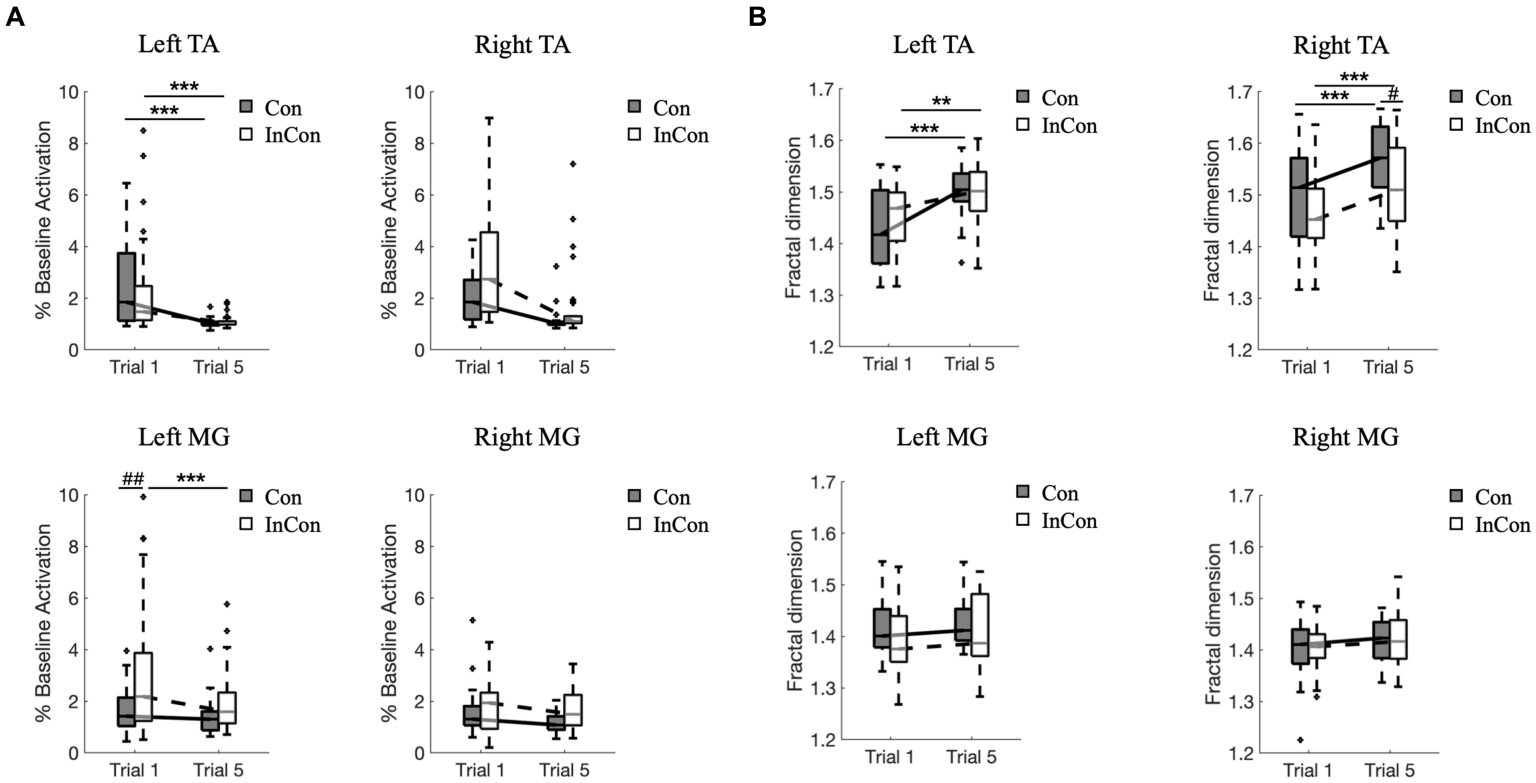
Changes of normalized muscle activity **(A)** and fractal dimension values of sEMG data **(B)** from left and right lower limbs. In each box plot, the box represents 1st and 3rd quartiles with the median value. The asterisk and the sharp indicate a significant difference (***<0.001, ##<0.01) between trials and groups, respectively. Con and InCon represent congruent and incongruent conditions, respectively. MG, medial gastrocnemius; TA, tibialis anterior.

A two-way mixed ANOVA revealed a significant main effect of practice on the FD values and on the normalized activity of TA and MG muscles in sEMG data. Also, there was a significant main effect of group on the FD values of right TA and on the normalized activity of right TA and left and right MG (Supplementary Table S2). However, no interaction effect of practice × group was observed. *Post-hoc* tests revealed that practice under the congruent and incongruent conditions increased FD values of sEMG in left and right TA muscles (*p* < 0.01) (Figure 3B), and decrease the sEMG activity in left TA muscle (*p* < 0.001) (Figure 3A). Besides, the FD value of right TA muscle in sEMG was greater under the 5th congruent condition compared with the 5th incongruent condition (*p* < 0.05) (Figure 3B). Left MG muscle activity was greater under the 1st incongruent then 1st congruent condition (*p* < 0.01) (Figure 3A).

### 3.3 Measures of EEG

**Figure 4:**
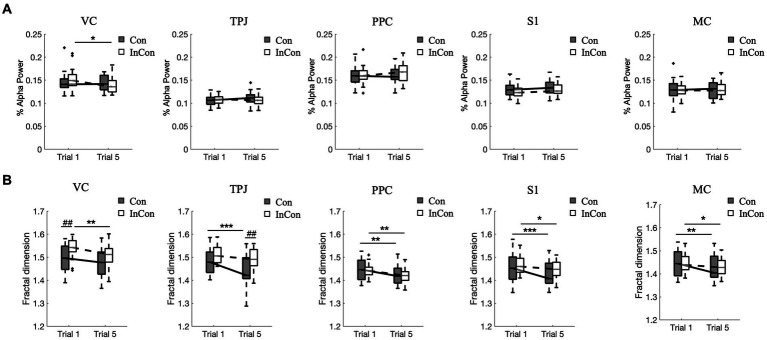
Changes of normalized alpha power values **(A)** and fractal dimension values of EEG data **(B)**. In each box plot, the box represents 1st and 3rd quartiles with the median value. The asterisk and the sharp (4) indicate a significant difference (*<0.05, **<0.01, ***<0.001, ##<0.01) between trials and groups, respectively. Con and lnCon represent congruent and incongruent conditions, respectively. MC, motor cortex; PPC, posterior parietal cortex; S1, primary somatosensory; TPJ, temporal-parietal junction; VC, visual cortex.

A two-way mixed ANOVA revealed a significant effect of practice on the FD values of VC, TPJ, PPC, S1 and MC in EEG and on the alpha-band power value of VC, and a significant effect of group on the FD values of VC and TPJ in EEG. However, no interaction effect of practice × group was observed (Supplementary Table S3). *Post-hoc* tests revealed that practice under the congruent condition decreased the FD values of EEG in MC, S1, PPC, and TPJ (*p* < 0.01) (Figure 4B), while practice under the incongruent condition decreased the FD values of EEG in MC, S1, PPC, and VC (*p* < 0.05) (Figure 4B) and decreased the alpha-band power value of VC (*p* < 0.05) (Figure 4A). Besides, the FD value of EEG in VC was greater under the 1st incongruent condition compared with the 1st congruent condition (*p* < 0.01), and the FD values of EEG in TPJ under the 5th incongruent condition was greater compared with the 5th congruent condition (*p* < 0.01) (Figure 4B).

### 3.4 Correlations

The two-tailed Pearson correlation tests found that (1) significant positive correlations between the complexity of high-frequency component of COP data in AP and ML directions and the complexity of sEMG data of left and right calf muscles (Table 1); (2) significant positive correlations between the complexity of low-frequency component of COP data in AP direction and the complexity of EEG data of VC, TPJ, and PPC (Table 2).

**Table 1:** Correlations between the complexity of high-frequency component of COP data and the complexity of sEMG data during visual-vestibular congruent and incongruent conditions (measured by FD values).

	Left TA	Left MG	Right TA	Right MG
COP_high_ ML	0.358***	0.219*	0.316***	0.145
COP_high_ AP	0.323***	0.246**	0.325***	0.190*

* Significant differences: *p* < 0.05; ** Significant differences: *p* < 0.01; ***Significant differences: *p* < 0.001.AP, anterior–posterior; FD, fractal dimension; MG, medial gastrocnemius; ML, medial-lateral; TA, tibialis anterior.

**Table 2:** Correlations between the complexity of low-frequency component of COP data and the complexity of EEG data of ROIs during visual-vestibular congruent and incongruent conditions (measured by FD values).

	VC	TPJ	PPC	S1	MC
COP_low_ ML	0.089	0.038	0.054	0.035	0.023
COP_low_ AP	0.208*	0.230*	0.214*	0.179	0.134

* Significant differences: *p* < 0.05.AP, anterior–posterior; FD, fractal dimension; MC, motor cortex; ML, medial-lateral; PPC, posterior parietal cortex; ROIs, regions of interest; S1, primary somatosensory; TPJ, temporal–parietal junction; VC, visual cortex.

## 4 Discussion

In this study, we analyze the dynamic changes in the cortical activity, calf muscle activity and postural sway after repeated balance practice under visual-vestibular congruent and incongruent environments by computing the linear measures and fractal exponent of EEG, sEMG and COP signals. We mainly found that (1) practice under congruent and incongruent conditions both increased the complexity of high-frequency fluctuations of COP data and the complexity of sEMG data of TA; (2) practice under incongruent condition decreased the complexity of low-frequency fluctuations of COP data and the complexity of EEG data of parietal and occipital lobes, whereas practice under congruent condition decreased the complexity of EEG signals of parietal and temporal lobes; (3) the complexity of high-frequency fluctuations of COP data was positively correlated with the complexity of sEMG data of calf muscles; (4) the complexity of low-frequency fluctuations of COP data was positively correlated with the complexity of EEG data of visual cortex and posterior parietal cortex. We believe that the use of complexity should be explored in future studies to investigate the underlying mechanisms of postural control.

### 4.1 Interpretation of complexity

Our fractal analysis of high-frequency components of COP signals revealed a significant increase after repeated balance practice under congruent and incongruent conditions (Figure 2B). This increased complexity of postural sway is in agreement with van den Hoorn et al. (2018), suggesting that increased complexity of postural sway indicates an improved ability to adapt to changing environments with effective strategies in postural control (Kędziorek and Blażkiewicz, 2020).

Our fractal analysis of sEMG signals showed a significantly increase in the fractal exponent of TA muscle after repeated practice under congruent and incongruent conditions (Figure 3B, top). According to these present results and previous findings that more unstable standing conditions induced lower sEMG complexity of calf muscles (Murillo et al., 2012), we suggest that higher complexity of sEMG data probably indicate the greater capacity of postural muscles to adapt to the perturbation. However, our present results showed no significant changes in the fractal exponent of MG muscle activity after repeated practice under congruent or incongruent condition (Figure 3B, bottom). Accordingly, we might suggest that TA muscles adapted to the perturbation in a more effective way than MG muscles. This is consistent with Schmid et al. (2011), who found that activity of TA decreased more significantly than that of MG during a 3-min backward and forward horizontal oscillations of the support base. It is reasonable to assume that muscles responsible for dorsiflexion (i.e., TA) and plantar flexion (i.e., MG) of the ankle play different role in postural control. Changes in muscle length of TA better reflect the changes in ankle angle, thus providing a better source of proprioceptive inputs; whereas MG actively contributes to stabilizing continuous postural sway (Merletti et al., 2010).

The changes in muscle activation and the complexity of sEMG signals are not always align across different tasks. For example, muscle fatigue decreased the muscle activity (%MVC) and also decreased the complexity of sEMG data with lower FD values or lower sample entropy values (Rampichini et al., 2020). However, our present results showed that practice under incongruent condition decreased the left TA muscles activity but increased the complexity of left TA sEMG data with higher FD values. This results are consistent with previous studies, where showed that when balance task difficulty increased, the calf muscle activity increased (Gebel et al., 2019) and the complexity of sEMG data decreased (Murillo et al., 2012; Pakniyat and Namazi, 2021). Besides, we also observed that practice under incongruent condition reduced the left MG muscle activity without any significant changes of fractal exponent of left MG muscle (Figure 3). Thus, when exploring changes in the sEMG data caused by different factors like muscle fatigue or postural perturbations, it is possible to comprehensively interpret the results from linear and nonlinear measures.

Although we found an increase in the fractal exponent of sEMG data from TA and high-frequency of COP data, the results of the analysis of EEG signals showed a reverse trend that repeated balance practice under congruent and incongruent conditions reduced the fractal exponent of EEG signals in different ROIs (Figure 4B). This reverse trend aligns with the findings in Pakniyat and Namazi (2021), where the complexity of EEG signals was higher in walking than standing in response to perturbation whereas the complexity of sEMG signals exhibited the opposite pattern, being higher in standing than walking. We could speculate that complexity environment might increase the complexity of EEG (Kamal et al., 2020; Pakniyat and Namazi, 2021). Accordingly, the decrease of complexity of EEG signals after habituation balance practice in the present study might correlate with the decrease of level of central volitional control or level of attention (Ming et al., 2009).

### 4.2 Relationship between COP and sEMG/EEG

However, our fractal analysis of low-frequency COP signals revealed a significant decrease after repeated practice under incongruent condition (Figure 2B). Moreno et al. (2022) recently argued that the long-time latency changes in low-frequency of COP signals align with the idea that regulating the COP to the desired state is primarily governed by alterations in the body’s reference configuration linked to central volitional control. Accordingly, the decreased complexity of low-pass components of COP suggested a decreased level of central volitional control after repeated practice under conflicting environments. Our correlation results are consistent with this hypothesis, showing that the complexity of low-frequency component of COP data was positively related with the complexity of EEG data from visual cortex and posterior parietal cortex (Table 2). Furthermore, we also found a significantly positive correlation between the complexity of high-frequency component of COP data and the complexity of sEMG data from calf muscles (Table 1). These results supported the previous findings that low- and high-frequency components of COP might be related to peripheral adjustments (such as reflex mechanisms and mechanical muscular properties) and central adjustments (such as cortical response), respectively (Zatsiorsky and Duarte, 1999; Moreno et al., 2022).

### 4.3 Cortical activity and sensory conflicts

Previous studies suggested that the increased complexity of EEG signals could reflect a good cortical response to stimuli (Takahashi et al., 2009). Accordingly, we observed that repeated practice under incongruent condition both reduced the fractal exponents of visual cortex EEG signals and the alpha-band power values of visual cortex (Figure 4). This result is consistent with the sensory reweighting theory (Peterka, 2002), suggesting that the brain probably down-weighted the less reliable visual information in our experimental setting for postural control. In contrast, our results showed a significantly decrease in the fractal exponents of TPJ EEG signals after repeated practice under congruent condition rather than incongruent condition, while there was no significant change of alpha-band power value after practice under congruent or incongruent condition (Figure 4). Although the superior temporal lobe has both congruent neurons and opposite neurons (Gu et al., 2006), the complexity of EEG data from superior temporal lobe reduced after repeated practice under congruent condition whereas the superior temporal lobe continuously played a key role in processing conflicting sensory information (Wang et al., 2022). These results suggested nonlinear dynamics of cortical activity differed after balance practice under congruent and incongruent environments.

### 4.4 Limitations

Since the present study aimed to investigate the effect of visual-vestibular conflicts on the dynamics of postural control, we chose the data from acceleration and deceleration phases, necessitating the stimulation of the vestibular system. Thus, one limitation is that we did not further explore the dynamic changes from other phases, such as baseline and recovery phases. However, it would be beneficial for further studies to investigate the dynamic changes after perturbations (i.e., the recovery phase) using complexity-based nonlinear methods, since previous studies showed that perturbations had a noticeable after-effect on postural control among young and older adults (Doumas and Krampe, 2010; van den Hoorn et al., 2018).

## 5 Conclusion

Our results showed the increased complexity of high-frequency component of COP after repeated balance practice under both congruent and incongruent conditions. The high-frequency component of COP might be related to mechanical muscular properties, as evidenced by our current findings showing an increased complexity of sEMG data of TA following balance practice, and also a significantly positive correlation between the complexity of high-frequency component of COP and the complexity of sEMG signals. The increased complexity of high-frequency component of COP and sEMG data after balance practice revealed a better ability of postural control system to be flexible in response to perturbations. On the other hand, our results showed the decreased complexity of low-frequency component of COP after repeated balance practice under incongruent condition. The low-frequency component of COP might be related to central mechanisms of postural control, as indicated by the present findings showing a reduced complexity of cortical activity of parietal and occipital lobes following balance practice, and also a significantly positive correlation between the complexity of low-frequency component of COP and the complexity of EEG signals. Accordingly, we believe that the use of complexity-based nonlinear measures should be explored in future studies to investigate the underlying mechanisms of the postural control.

## Data availability statement

The raw data supporting the conclusions of this article will be made available by the authors, without undue reservation.

## Ethics statement

The studies involving humans were approved by The Ethics Committee of Zhejiang University Psychological Science Research Center. The studies were conducted in accordance with the local legislation and institutional requirements. The participants provided their written informed consent to participate in this study. Written informed consent was obtained from the individual(s) for the publication of any potentially identifiable images or data included in this article.

## Author contributions

AH: Data curation, Formal analysis, Methodology, Writing – original draft. GW: Data curation, Software, Writing – review & editing. JB: Formal analysis, Methodology, Writing – review & editing. ZH: Methodology, Software, Writing – review & editing. JL: Formal analysis, Visualization, Writing – review & editing. JM: Formal analysis, Investigation, Visualization, Writing – review & editing. JW: Conceptualization, Investigation, Project administration, Supervision, Writing – review & editing.
