# Supplementary material for: Nonlinear dynamics of postural control system under visual-vestibular habituation balance practice: evidence from EEG, EMG and center of pressure signals
Source: Front Hum Neurosci. 2024 Apr 26;18:1371648. doi: 10.3389/fnhum.2024.1371648 (PMC11082324; doi:10.3389/fnhum.2024.1371648)
Supplement: Supplementary file 1 [file Data_Sheet_1.docx]

Table S1. Two-way mixed ANOVA of SD values and FD values of low- and high-frequency variation in COP data by training and Group factors.

|  |  | *F_(1,52)_* | *p* value | *η²* |
| --- | --- | --- | --- | --- |
| SD  (AP direction) | Training  Group  Training$\times$Group | **5.352**  3.730  0.463 | **<0.05**  0.06  0.49 | **0.09** |
| SD  (ML direction) | Training  Group  Training$\times$Group | **8.940**  **4.110**  0.171 | **<0.01**  **<0.05**  0.68 | **0.15**  **0.07** |
| FD _low_  (AP direction) | Training  Group  Training$\times$Group | **13.880**  2.236  0.807 | **<0.001**  0.14  0.37 | **0.21** |
| FD _low_  (ML direction)  FD _high_  (AP direction) | Training  Group  Training$\times$Group  Training  Group  Training$\times$Group | **4.653**  0.028  3.383  **73.431**  1.162  0.269 | **<0.05**  0.98  0.07  **<0.001**  0.28  0.60 | **0.08**  **0.58** |
| FD _high_  (ML direction) | Training  Group  Training$\times$Group | **71.320**  0.908  0.034 | **<0.001**  0.34  0.95 | **0.57** |

ANOVA main effects and interactions are presented as *F* score and *p* value. Effect sizes were calculated using partial eta squared (*η²*). AP, anterior-posterior; COP, center of pressure; FD, fractal dimension (low, low-frequency of COP; high, high-frequency of COP); ML, medial-lateral; SD, standard deviation.

Table S2. Two-way mixed ANOVA of normalized activity values and FD values of muscles in sEMG data by training and Group factors.

|  |  | *F_(1,52)_* | *p* value | *η²* |
| --- | --- | --- | --- | --- |
| Activity  (Left TA)  Activity  (Right TA)  Activity  (Left MG)  Activity  (Right MG)  FD  (Left TA) | Training  Group  Training$\times$Group  Training  Group  Training$\times$Group  Training  Group  Training$\times$Group  Training  Group  Training$\times$Group  Training  Group  Training$\times$Group | **33.970**  0.014  0.085  **42.511**  **7.242**  2.463  **12.173**  **8.693**  3.715  **6.825**  **4.199**  1.998  **39.220**  0.251  3.905 | **<0.001**  0.97  0.76  **<0.001**  **<0.01**  0.12  **<0.01**  **<0.01**  0.06  **<0.05**  **<0.05**  0.16  **<0.001**  0.61  0.05 | **0.39**  **0.45**  **0.12**  **0.19**  **0.14**  **0.11**  **0.07**  **0.43** |
| FD  (Right TA)  FD  (Left MG) | Training  Group  Training$\times$Group Training  Group  Training$\times$Group | **38.771**  **5.167**  0.310  **6.856**  1.202  0.140 | **<0.001**  **<0.05**  0.57  **<0.05**  0.27  0.70 | **0.42**  **0.10**  **0.12** |
| FD  (Right MG) | Training  Group  Training$\times$Group | **6.568**  0.013  0.060 | **<0.05**  0.90  0.93 | **0.11** |

ANOVA main effects and interactions are presented as *F* score and *p* value. Effect sizes were calculated using partial eta squared (*η²*). FD, fractal dimension; MG, medial gastrocnemius; sEMG, surface electromyography; TA, tibialis anterior.

Table S3. Two-way mixed ANOVA of FD values of ROIs in EEG data by training and Group factors.

|  |  | *F_(1,52)_* | *p* | *η²* |
| --- | --- | --- | --- | --- |
| VC | Training  Group  Training$\times$Group | **15.520**  **9.477**  1.269 | **<0.001**  **<0.01**  0.26 | **0.23**  **0.15** |
| TPJ  PPC  S1  MC  FEF  DL-PFC | Training  Group  Training$\times$Group  Training  Group  Training$\times$Group  Training  Group  Training$\times$Group  Training  Group  Training$\times$Group  Training  Group  Training$\times$Group  Training  Group  Training$\times$Group | **18.790**  **9.972**  1.845  **21.671**  0.053  0.018  **21.220**  1.581  0.335  **14.072**  0.039  0.133  2.947  1.423  1.814  1.346  1.117  0.046 | **<0.001**  **<0.01**  0.18  **<0.001**  0.81  0.89  **<0.001**  0.21  0.56  **<0.001**  0.84  0.71  0.09  0.23  0.18  0.25  0.29  0.82 | **0.26**  **0.16**  **0.29**  **0.28**  **0.21** |

ANOVA main effects and interactions are presented as *F* score and *p* value. Effect sizes were calculated using partial eta squared (*η²*). DL-PFC, dorsolateral prefrontal cortex; EEG, electroencephalogram; FEF, frontal eye field cortex; MC, motor cortex; PPC, posterior parietal cortex; ROIs, regions of interest; S1, primary somatosensory; TPJ, temporal-parietal junction; VC, visual cortex.

Table S4. Two-way mixed ANOVA of alpha-band power values of ROIs in EEG data by training and Group factors.

|  |  | *F_(1,52)_* | *p* | *η²* |
| --- | --- | --- | --- | --- |
| VC | Training  Group  Training$\times$Group | **7.746**  0.139  2.065 | **<0.01**  0.71  0.15 | **0.13** |
| TPJ  PPC  S1  MC  FEF  DL-PFC | Training  Group  Training$\times$Group  Training  Group  Training$\times$Group  Training  Group  Training$\times$Group  Training  Group  Training$\times$Group  Training  Group  Training$\times$Group  Training  Group  Training$\times$Group | 0.273  0.732  4.780  1.166  0.462  0.028  **5.325**  3.110  0.520  0.186  0.047  0.701  0.32  0.02  0.76  0.065  1.331  0.102 | 0.60  0.39  0.05  0.28  0.49  0.86  **<0.05**  0.08  0.47  0.66  0.83  0.40  0.56  0.89  0.38  0.79  0.25  0.75 | **0.09** |

ANOVA main effects and interactions are presented as *F* score and *p* value. Effect sizes were calculated using partial eta squared (*η²*). DL-PFC, dorsolateral prefrontal cortex; EEG, electroencephalogram; FEF, frontal eye field cortex; MC, motor cortex; PPC, posterior parietal cortex; ROIs, regions of interest; S1, primary somatosensory; TPJ, temporal-parietal junction; VC, visual cortex.
